# Supplementary material for: Estimating disease prevalence in large datasets using genetic risk scores
Source: Nat Commun. 2021 Nov 8;12:6441. doi: 10.1038/s41467-021-26501-7 (PMC8575951; doi:10.1038/s41467-021-26501-7)
Supplement: Supplementary file 1 — Supplementary Information [file 41467_2021_26501_MOESM1_ESM.pdf]

Supplementary notes and figures for:
Estimating disease prevalence in large datasets using genetic risk scores

Benjamin D. Evans[1,2,3,*], Piotr Słowiński[1,4,*], Andrew T. Hattersley[5,6], Samuel E. Jones[5], Seth Sharp[5], Robert A. Kimmitt[5,6] Michael N. Weedon[5], Richard A. Oram[5,6], Krasimira Tsaneva-Atanasova[1,7], Nicholas J. Thomas[1,2,6]

1. Department of Mathematics, University of Exeter, North Park Road, Exeter, EX4 4QF, UK.
2. Living Systems Institute, Centre for Biomedical Modelling and Analysis, University of Exeter, Stocker Road, Exeter, EX4 4QD, UK.
3. School of Psychological Science, University of Bristol, Priory Road, Bristol, BS8 1TU, UK.
4. Living Systems Institute, Translational Research Exchange @ Exeter, University of Exeter, Stocker Road, EX4 4QD, UK.
5. University of Exeter Medical School. Address: Institute of Biomedical & Clinical Science, RILD Building, Royal Devon & Exeter Hospital, Barrack Road, Exeter EX2 5DW, UK.
6. Royal Devon & Exeter NHS Foundation Trust, Exeter, UK.
7. Living Systems Institute, EPSRC Hub for Quantitative Modelling in Healthcare, University of Exeter, Stocker Road, EX4 4QD, UK.

* Denotes equal contribution.

Corresponding author
Nicholas Thomas

Institute of Biomedical and Clinical Science & NIHR Exeter Clinical Research Facility,

University of Exeter Medical School, Exeter, UK

Email: n.thomas3@exeter.ac.uk

Tel: **01392408325**

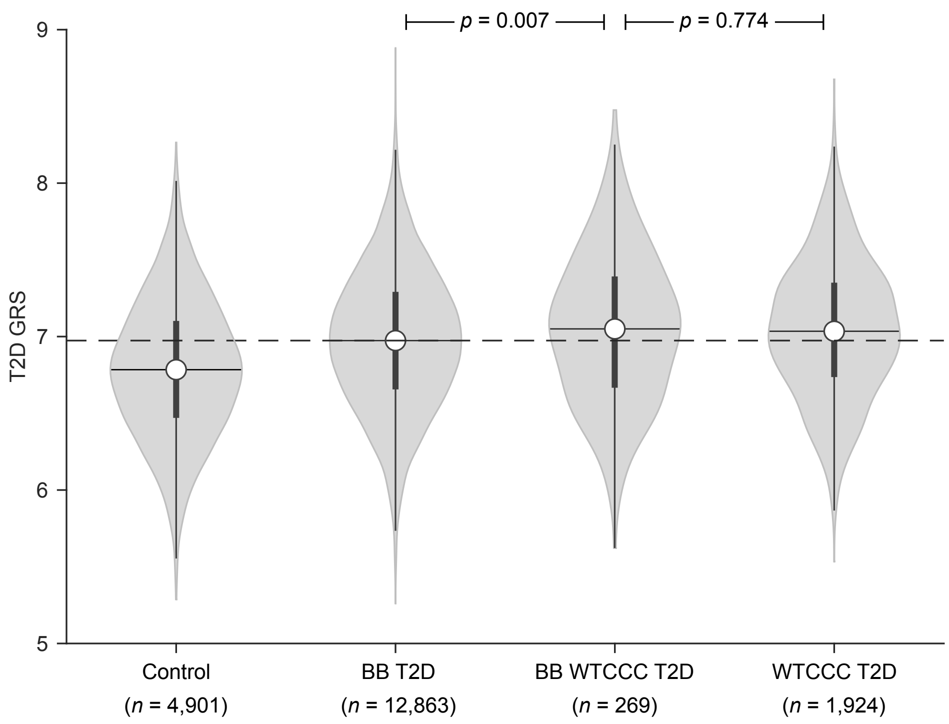

Supplementary Figure 1: Illustration of the effect of non-preserved equivalence in different dataset. BB T2D is a Biobank Type 2 diabetes cohort defined as diabetes diagnosed over 30 years of age and non-insulin treated. BB WTCCC T2D cohort is defined as first degree relative with type 1 diabetes diagnosed over 30 up to 40 years of age to recreate the WTCCC cohort. The WTCCC T2D cohort is also shown. p-values are from two-tailed t-tests and indicate that GRS of BB WTCCC T2D and WTCCC T2D cohorts have the same mean value, that is different from the mean of BB T2D cohort. The outline shows kernel density estimate of the GRS distribution (outlines end at the maxima and minima of the samples). The boxplots show median (circle), the box (thick vertical line) extends from the 25th to the 75th percentile, the whiskers (thin vertical line) extend by 1.5 IQR from the box. Thin horizontal lines indicate means, dashed horizontal line indicates mean GRS of the BB T2D cohort.

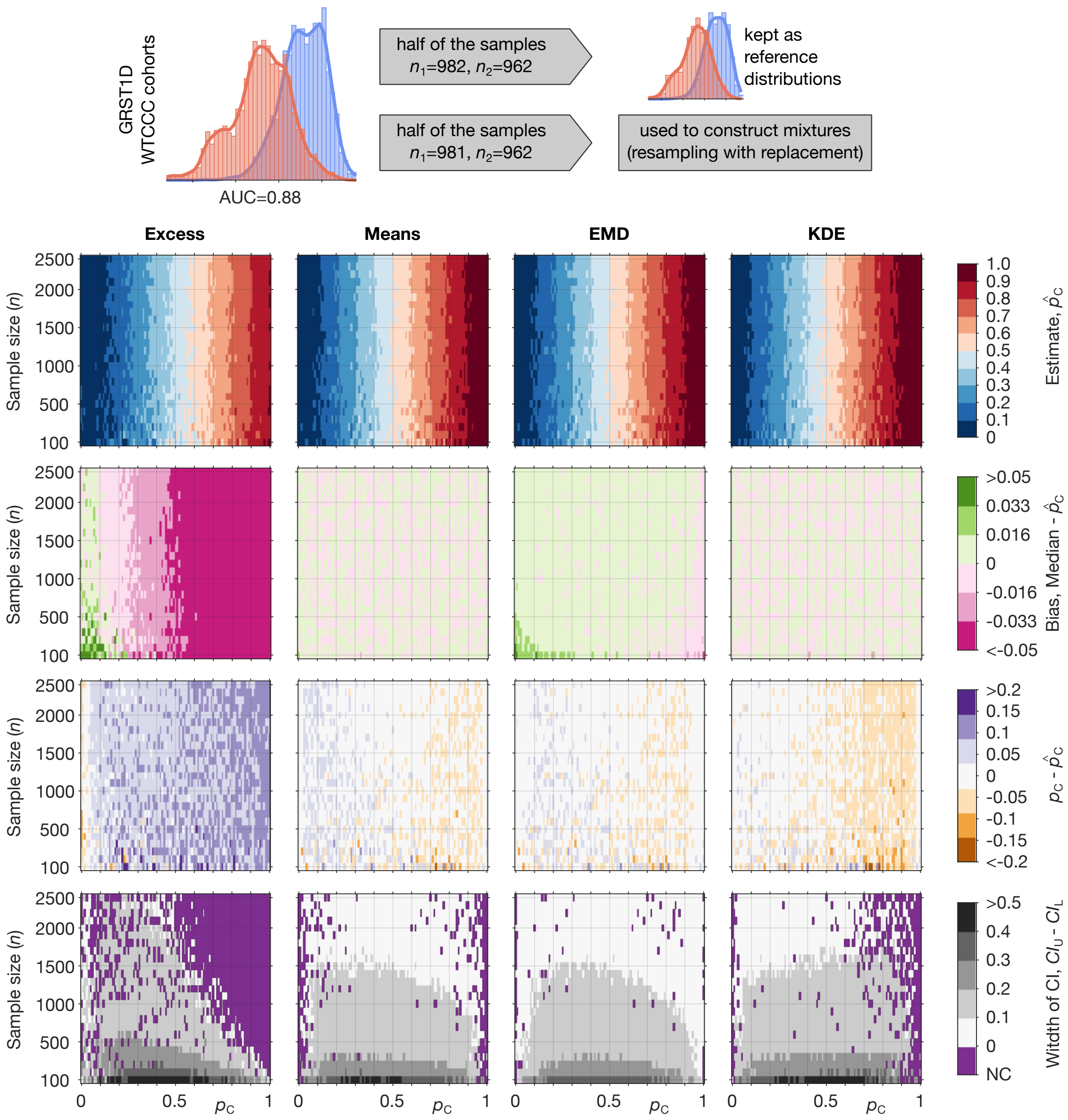

Supplementary Figure 2: A comparison of the four methods prevalence estimates and confidence intervals for varying proportion of disease and cohort sizes using the T1DGRS from the WTCCC dataset ($n = 3,887$). (Top row) Illustration of how the reference samples (WTCCC dataset) were divided into subsamples. Half of each reference sample was used as a reference distribution and the other half was used to construct mixture samples. (Second row) Estimate of prevalence ($\hat{p}_C$) in the constructed mixtures. (Third row) Bias of the prevalence estimates ($\hat{p}_C$) across the constructed mixtures. (Fourth row) deviation from the true proportion ($p_C - \hat{p}_C$) across the constructed mixtures. (Bottom row) The width of confidence ($CI_U - CI_L$) intervals of the estimates across the constructed mixtures. The purple colour (bottom row) indicates regions in which the confidence interval did not include the true value (p_C), $CI_U = CI_L$ or CI are undefined (both latter cases can happen if $\hat{p}_C = 0$ or $\hat{p}_C = 1$). Sample sizes: R_C – cases WTCCC type 1 diabetes ($n = 982$), R_N – non-cases WTCCC Type 2 diabetes ($n = 962$), mixtures – sampled with replacement from a holdout half of the R_C ($n = 981$) and R_N ($n = 962$) samples.

Supplementary Note 1: Quantitative characterisation of methods

To compare bias and precision (quantified as the width of CI) of all the methods we compared their performance assuming that $p_{\mathrm{C}} = \hat{p}_{\mathrm{C}}$ and acceleration=0. Again, the proportion and sample size were systematically varied, with p_{C} ranging from 0 to 1 in 0.01 (1%) steps while n ranged from 100 to 2,500 in steps of 100 samples. All four methods were applied to each combination of these parameters. At each point in the parameter space, we estimated the bias and confidence intervals. This idealised scenario allows a direct head-to-head comparison of accuracy between all four methods without the randomness originating from the sampling process. Results of this comparison are presented in Supplementary Figs 3-4.

Supplementary Figs 3-4 show:

- (1st row) Median of the 100,000 bootstrap samples $\mathrm{med}(\{\{p'_{\mathrm{C}}\}_{1000}\}_{100})$,
- (2nd row) Median bias $\mathrm{B} = \mathrm{med}(\{\{p'_{\mathrm{C}}\}_{1000}\}_{100}) - \hat{p}_{\mathrm{C}}.$,
- (3rd row) The width of confidence ($CI_{\mathrm{U}} - CI_{\mathrm{L}}$) intervals,

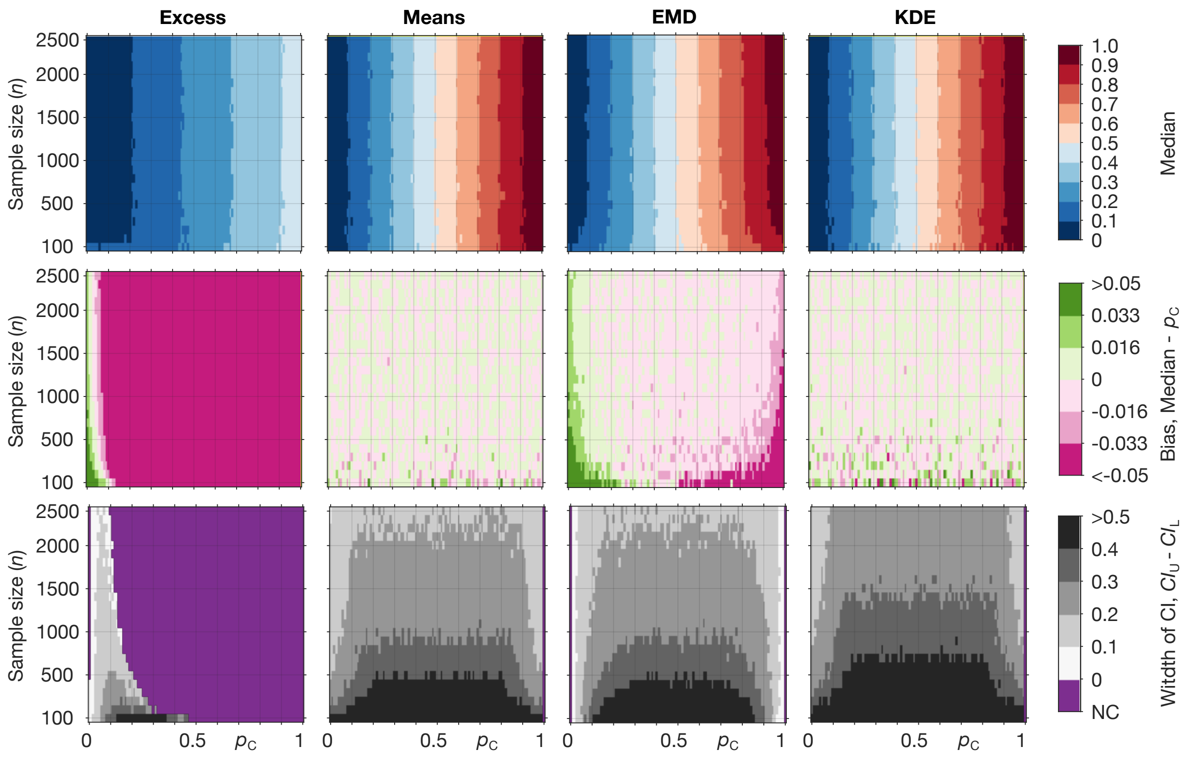

Supplementary Figure 3: Evaluation of the four Estimation Methods using T2DGRS from the WTCCC dataset ($n = 3,887$). (Top row) Median value of the 100,000 estimates of prevalence (p'_C) in the bootstrap samples across defined mixture proportions (p_C) and the mixture sample size (n) of the dataset for each method. (Second row) Median bias of the methods across the constructed samples. (Bottom row) The width of confidence intervals ($CI_U - CI_L$) of the individual estimates across the defined mixture proportions (p_C) and the mixture sample size (n). The purple colour (row 3) indicates regions in which the confidence interval did not include the true value (only observed for the Excess method), $CI_U = CI_L$ or CI are undefined (both latter cases can happen if $p_C = 0$ or $p_C = 1$). It can be observed that across the parameter space, the Means, EMD and KDE methods all typically outperform the Excess method. It is also evident that the Means and KDE methods practically do not exhibit any bias. A further increase of sample sizes would be recommended to reduce the width of the CI below 10% (see Table 1). Sample sizes: $\mathrm{R_C}$ – cases WTCCC type 1 diabetes ($n = 982$), $\mathrm{R_N}$ – non-cases WTCCC Type 2 diabetes ($n = 962$), mixtures – sampled with replacement from a holdout half of the $\mathrm{R_C}$ ($n = 981$) and $\mathrm{R_N}$ ($n = 962$) samples.

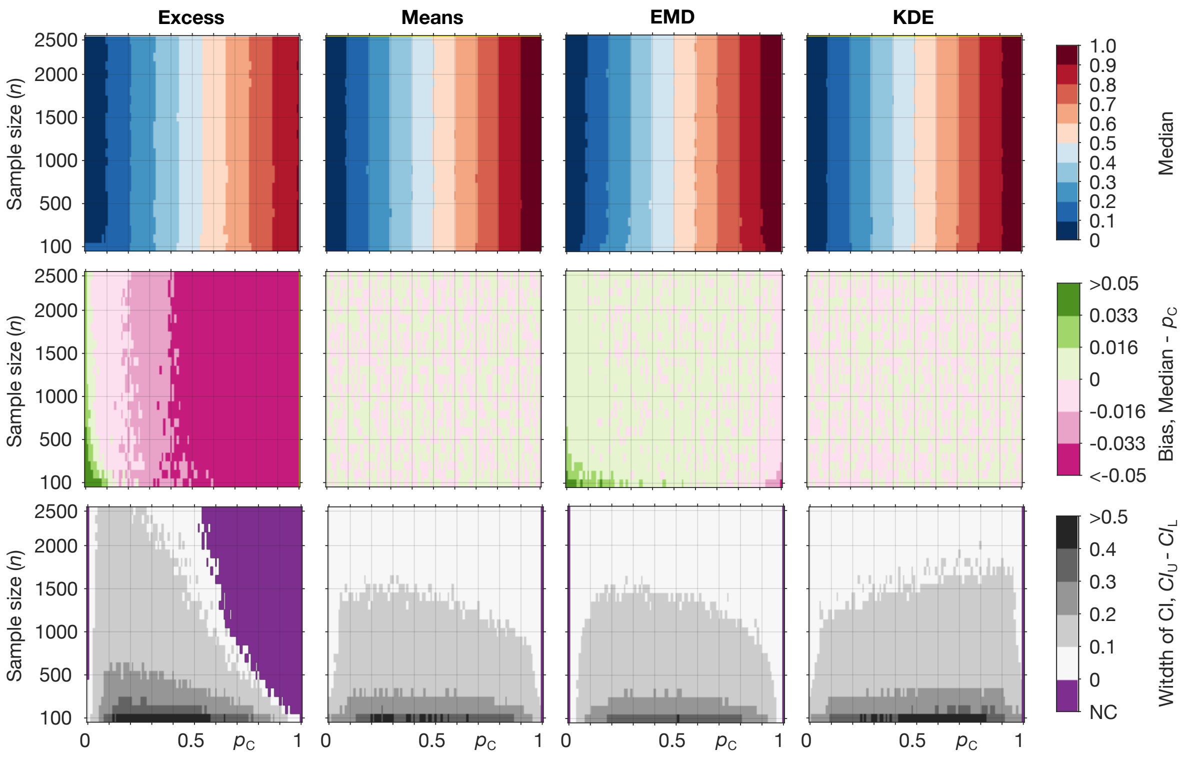

Supplementary Figure 4: Evaluation of four Estimation Methods using the T1DGRS from the WTCCC dataset ($n = 3,887$). (Top row) Median value of the 100,000 estimates of prevalence (p'_C) in the bootstrap samples across defined mixture proportions (p_C) and the mixture sample size (n) of the dataset for each method. (Second row) Median bias of the methods across the constructed samples. (Bottom row) The width of confidence intervals ($CI_U - CI_L$) of the individual estimates across the defined mixture proportions (p_C) and the mixture sample size (n). The purple colour (row 3) indicates regions in which the confidence interval did not include the true value (only observed for the Excess method), $CI_U = CI_L$ or CI are undefined (both latter cases can happen if $p_C = 0$ or $p_C = 1$). It can be observed that across the parameter space, the Means, EMD and KDE methods all typically outperform the Excess method. Sample sizes: R_C – cases WTCCC type 1 diabetes ($n = 982$), R_N – non-cases WTCCC Type 2 diabetes ($n = 962$), mixtures – sampled with replacement from a holdout half of the R_C ($n = 981$) and R_N ($n = 962$) samples.

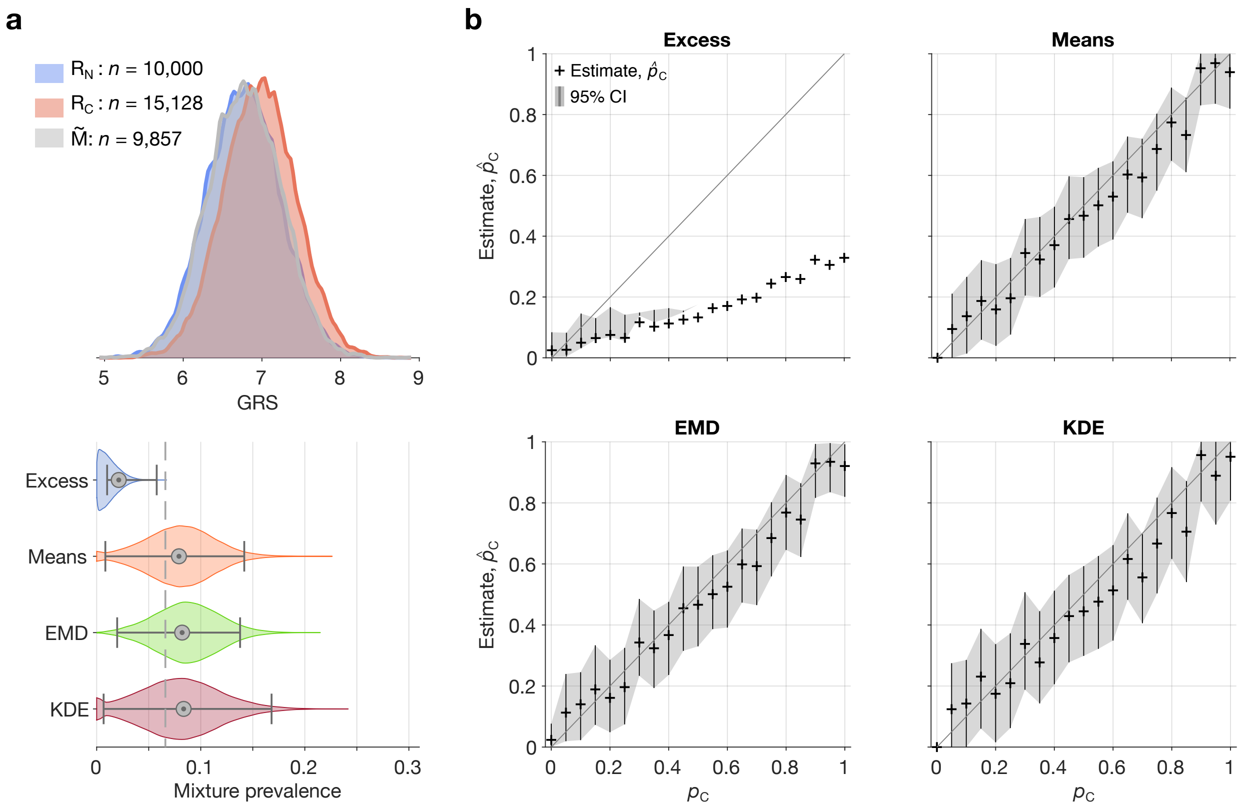

Supplementary Figure 5: Panel (a) illustrates a worked example using T2DGRS to estimate non-insulin treated diabetes cases within a cohort with glaucoma. All three new methodologies provide robust estimates of the proportion of individuals with type 2 diabetes with their 95% CIs (square brackets) encompassing the known proportion of 6.6%: Means = 7.9% [0.8%, 14.2%], EMD = 8.2% [2.0%, 13.8%], KDE = 8.4% [0.7%, 16.8%]. The 6.6% ground truth value was estimated using the reported number of type 2 diabetes cases within the glaucoma cohort in UK Biobank. Conversely the Excess method performs poorly (2.1% [1%, 5.8%]) and does not capture the known proportion. Cases of self-reported glaucoma (*n* = 9,857) were taken from unrelated individuals of white European descent in the UK Biobank. As estimating cases of type 2 diabetes analysis was restricted to exclude insulin treated diabetes cases. Diabetes was defined as self-reported or HbA1C ≥48 mmol mol^{-1} to identify undiagnosed cases. Reference controls used were white European participants without diabetes and glaucoma and cases were non-insulin treated diabetes cases from UK Biobank without glaucoma. Top, the reference and the mixture distributions ($\mathbf{R_C}$, shaded red, $\mathbf{R_N}$, shaded blue, $\widetilde{\mathbf{M}}$, shaded grey, respectively). Bottom, the estimated values of prevalence $\hat{p}_C$ (grey bullseyes) and 95% confidence intervals (horizontal lines with vertical bars at the ends). The violin plots show the distribution of the 100,000 estimates of prevalence (p'_C) in the bootstrap samples. Vertical dashed line indicates the known proportion of 6.6%. Panel (b) illustrates an experiment to evaluate the assumption that the GRS distribution in the mixture cohort $\widetilde{\mathbf{M}}$ (the glaucoma cases) is only affected by T2D prevalence. Using the GRS of the mixture cohort, $\widetilde{\mathbf{M}}$ ($n = 9,857$), we constructed 21 samples ($n = 2,500$, each) with prevalence of T2D varying from 0 to 100% (with 5% steps); sampling (with replacement) from the T2D cases and non-cases in the mixture cohort, $\widetilde{\mathbf{M}}$. For each of the constructed samples we computed an estimate of T2D prevalence (+ marker) and its confidence intervals (black vertical line and shading). The Means, EMD and KDE methods, return accurate estimates for T2D proportions varying from 0 to 100%. For Means and KDE method, the prevalence estimate for $p_C = 0$ is assumed to be $\hat{p}_C = 0$ (sample mean is smaller than both means of the reference samples and KDE estimate is <0).

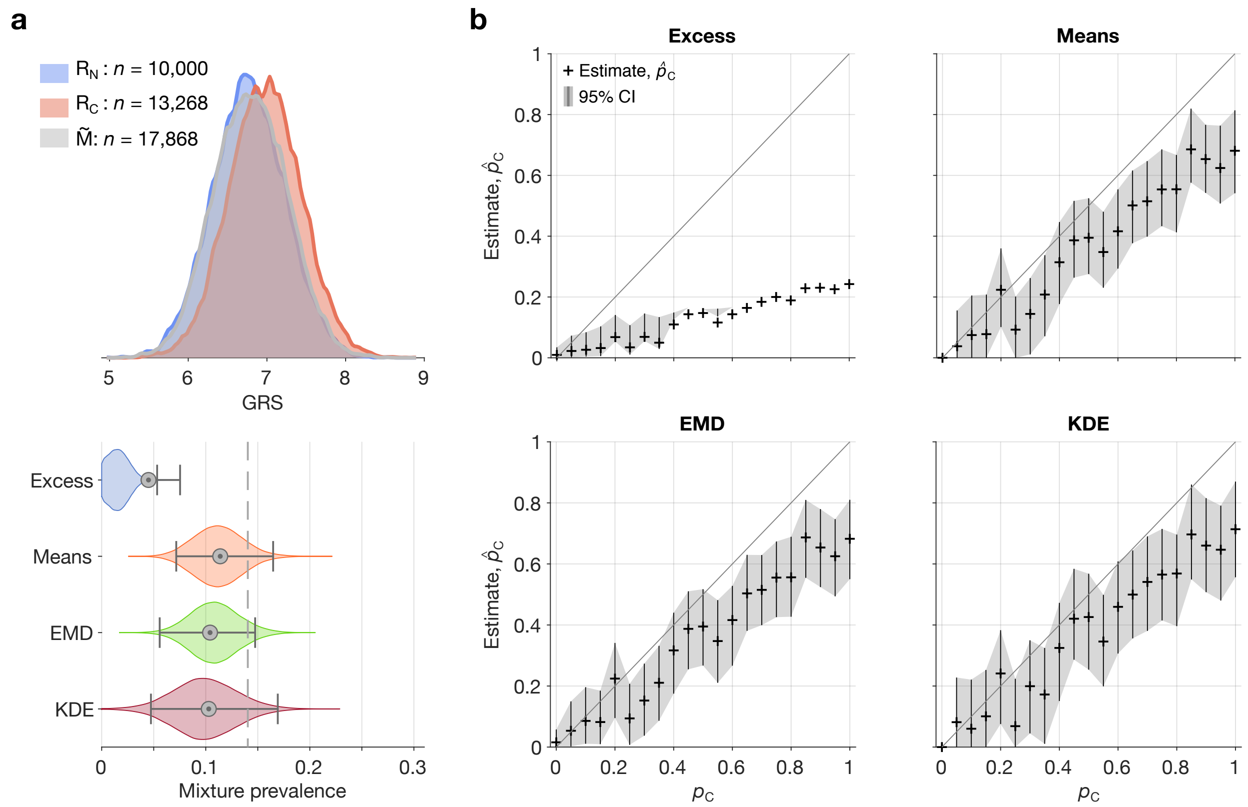

Supplementary Figure 6: Panel (a) illustrates a worked example using T2DGRS to estimate non-insulin treated diabetes cases within a cohort with microalbuminuria. All three new methodologies provide robust estimates of the proportion of individuals with type 2 diabetes with their 95% CIs (square brackets) encompassing the known proportion of 14.04%: Means = 11.4% [7.2%, 16.5%], EMD = 10.4% [5.6%, 14.8%], KDE = 10.3% [4.7%, 16.9%]. The 14.04% ground truth value was estimated using the reported number of type 2 diabetes cases within the microalbuminuria cohort in UK Biobank. Conversely the Excess method performs poorly (4.5% [5.3%, 7.5%]) and does not capture the known proportion. Markings and colours are the same as in the Supplementary Fig. 5. Panel (b) illustrates an experiment to evaluate the assumption that the GRS distribution in the mixture cohort $\tilde{M}$ (the microalbuminuria cases) is only affected by T2D prevalence. Methods and plots are the same as in the Supplementary Fig. 5. We observe that at higher proportions of T2D the performance of the methods reduces so under-estimates are returned. This reflects the fact that in T2D cases there is a subtle reduction in mean T2DGRS in those with microalbuminuria (6.93 (SD 0.45)) compared to those without (6.98 (SD 0.46)). This result may reflect collider bias because the microalbuminuria phenotype reflects a cohort with higher multifactorial environmental risk for T2D so T2D occurs with less T2D genetic predisposition. There is no difference in mean T2DGRS in non-T2D cases with (6.77 (SD 0.46)) or without microalbuminuria (6.77 (SD 0.46)). This explains the good performance for small T2D proportions.

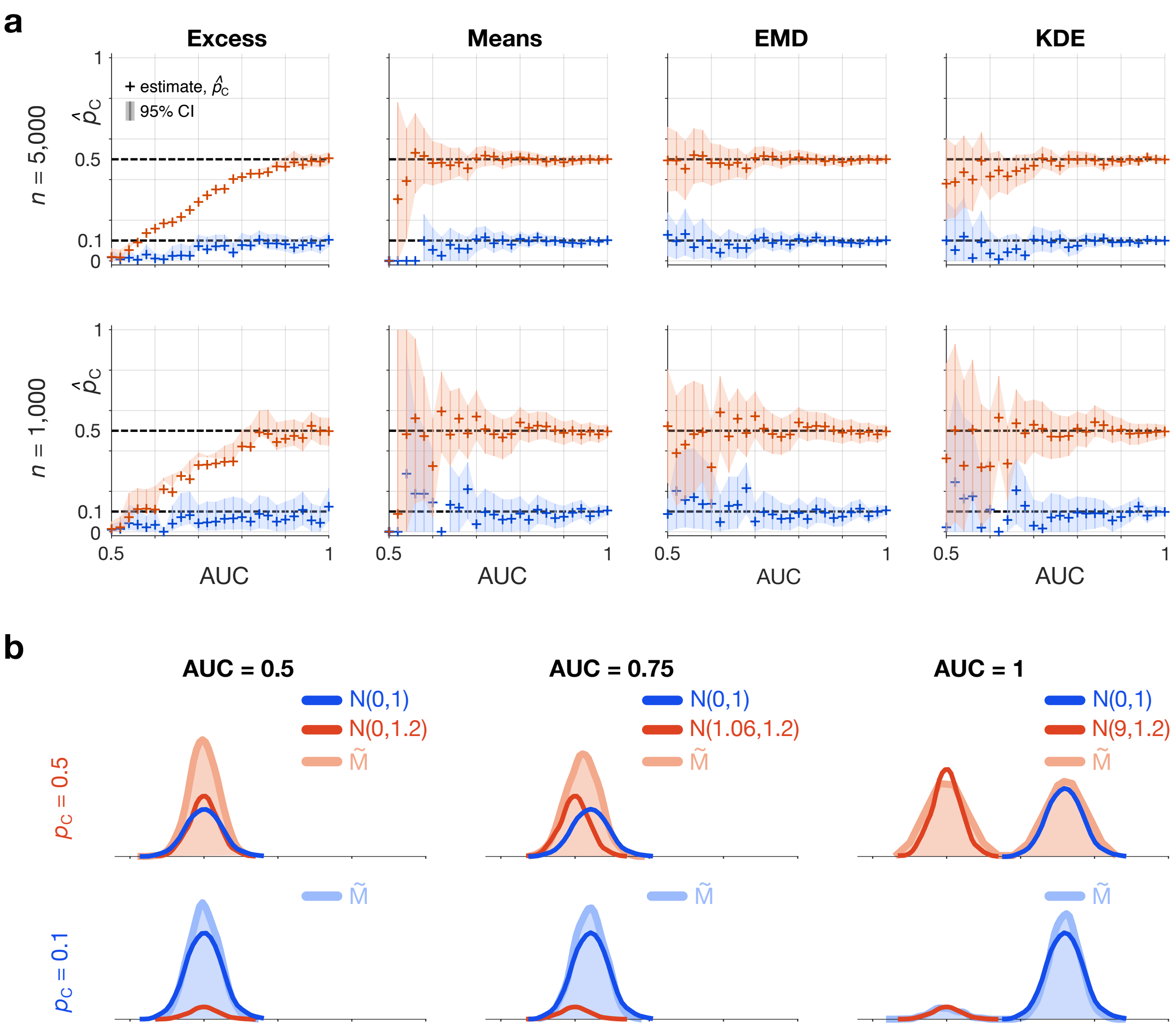

Supplementary Figure 7: A comparison of the four methods using an artificial genetic risk score with increasing discriminative ability as measured by AUC, from AUC = 0.5 (no discriminative ability) through to AUC = 1 (complete differentiation). (a) The estimated proportion (+ marker) with confidence intervals (vertical lines and shading) for each of the methods (Excess, Means, EMD, KDE) are shown using mixture sample size, $n = \{1000, 5000\}$. (b) Examples of the mixture distributions for AUC $= \{0.5, 0.75, 1\}$. N(μ,σ) is a normal distribution with mean μ and standard deviation σ and $\tilde{\mathrm{M}}$ is a mixture of the two normal distributions; $p_C = 0.5$ (pale red) or $p_C = 0.1$ (light blue). Reference distributions are indicated in red and blue. At several small AUC values, mean of the constructed mixture samples was smaller than both means of the reference samples, in these cases the prevalence estimate from the Means method is assumed to be $\hat{p}_C = 0$ and confidence intervals are undefined due to undetermined acceleration value. This figure is generated using artificial data. To generate reference samples with AUC varying from 0.5 to 1 with 0.02 step, one of the reference samples (blue) was fixed, sampled from N(0,1), the other reference sample (red) was sampled from normal distribution from N(0,1.2), and had its mean was shifted, by adding a constant, to the following values μ = {0.0, 0.093, 0.17, 0.25, 0.33, 0.41, 0.49, 0.57, 0.65, 0.4, 0.82, 0.91, 1.01, 1.1, 1.21, 1.31, 1.43, 1.55, 1.68, 1.83, 2, 2.17, 2.42, 2.73, 3.2, 9}. Both reference samples have $n = 2000$.

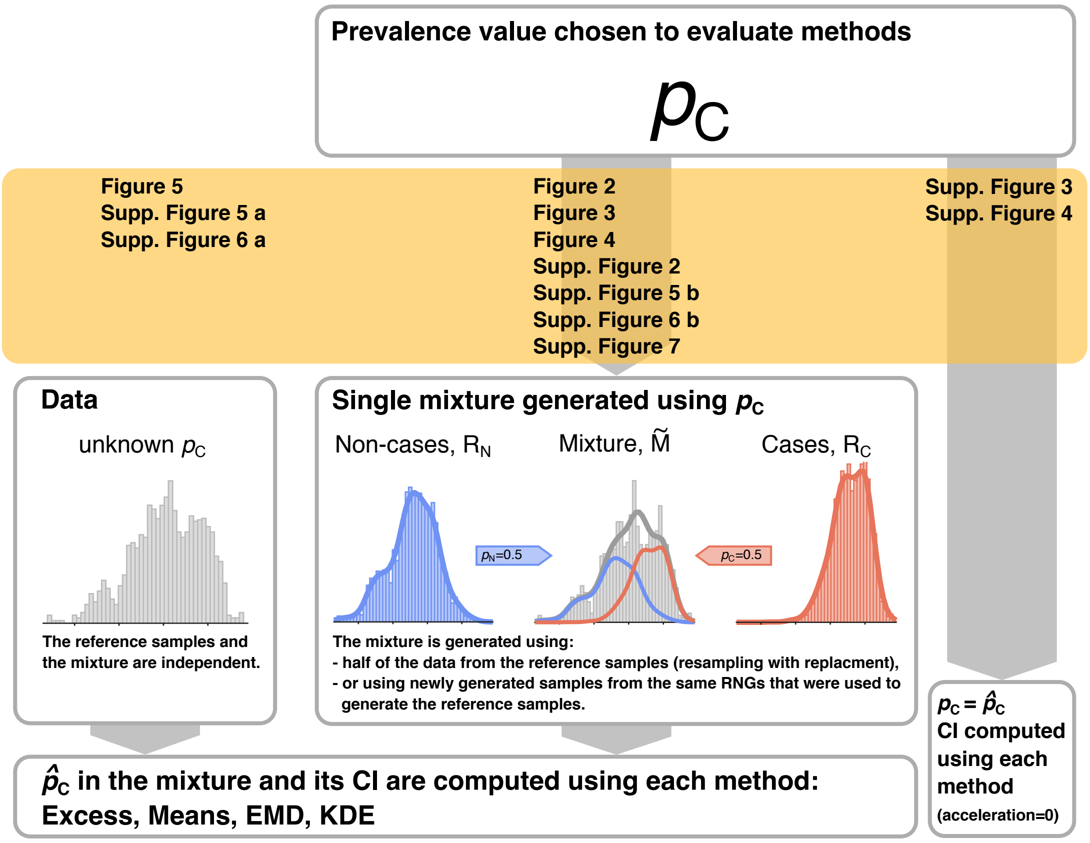

Supplementary Figure 8: Illustration of the approaches used to estimate $\hat{p}_C$ throughout the paper. The $\hat{p}_C$ can be estimated from data (real or simulated) or can be fixed by hand. We used simulated data generated with a specified value of p_C to evaluate the methods by comparing the estimated value, $\hat{p}_C$, with the true prevalence, p_C (results illustrated in Figs 3, 4 and 5, Supplementary Figs 2 and 5-7). In Supplementary Figs 3 and 4 with assume that $p_C = \hat{p}_C$. In this way we compare bias and width of the CI of the methods without the random effects caused by simulating mixture data.

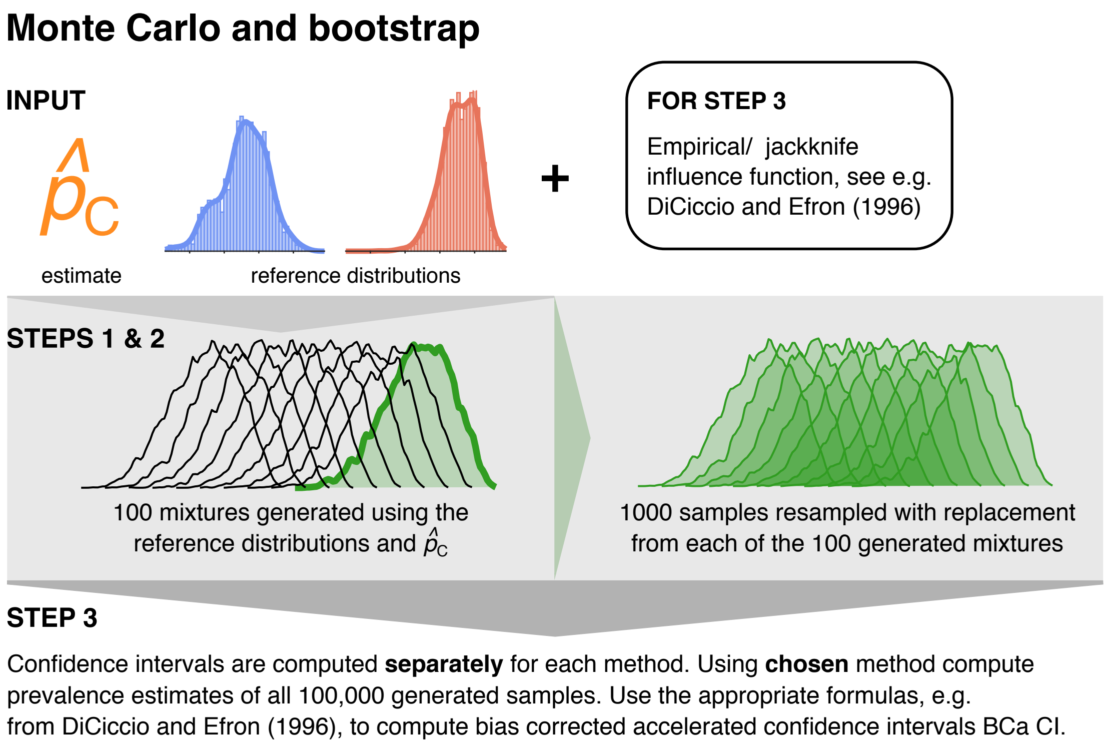

Supplementary Figure 9: Illustration of the steps used in estimation of the confidence intervals of the prevalence estimate $\hat{p}_C$. To find bias corrected and accelerated (BCa) confidence intervals, we used the estimate $\hat{p}_C$, sample size of the original mixture sample, and the reference samples. We generated $N_M = 100$ mixture samples with a given composition ($\hat{p}_C$) and sample size (n) equal to the size of the original mixture sample. Next, we resampled (with replacement) each of the $N_M = 100$ new mixtures, generating $N_B = 1,000$ bootstrap samples for each. We applied each chosen method to all generated samples to obtain $N_M \cdot N_B = 100,000$ bootstrapped estimates p_C'. We then used the methods described in section Calculating confidence intervals to find BCa confidence intervals.

Supplementary Note 2: The pure mixture assumption

To check if the mixture assumption, $p_{\mathrm{C}} + p_{\mathrm{N}} = 1$, is satisfied, three different errors of the point estimates of prevalence derived from the EMD and KDE methods, $e_{\mathrm{EMD,1}}$, $e_{\mathrm{EMD,2}}$, e_{KDE} could be further analysed. The $e_{\mathrm{EMD,1}}$ error captures the deviation of the mixture from the convex combination of the two reference samples. The $e_{\mathrm{EMD,2}}$ error is the EMD between the mixture and a model cumulative density function (CDF) based on the two independently estimated not normalised prevalence values $p_{\mathrm{C}}^{\mathrm{EMD}}$ and $p_{\mathrm{N}}^{\mathrm{EMD}}$. The e_{KDE} error is the sum of squared residuals (multiplied by the Gaussian kernels bandwidth) from the least-squares fitting procedure, which forms part of the KDE method. To interpret the initial point values of the errors, we compared them to 100,000 bootstrapped error values (as in all other computations we used 100 mixtures * 1,000 bootstraps). The bootstrap samples are generated using the two reference samples and their composition is based on the estimate of prevalence. In this way, we compare the error value of the investigated mixture sample with 100,000 values from a model that explicitly assumes there are only two reference populations (i.e., $p_{\mathrm{C}} + p_{\mathrm{N}} = 1$). This approach allows us to check how likely the occurrence of the observed error value is in the model for a given sample size and reference samples. The obtained bootstrap *p*-values are the number of bootstrapped modelled errors that are higher than the sample error and can be interpreted as the probability that the observed values of $e_{\mathrm{EMD,1}}$, $e_{\mathrm{EMD,2}}$ and e_{KDE} are a result of the sampling error. The bootstrap *p*-values are equivalent to *p*-values of a traditional statistical test (7, 11).

Supplementary Figure 10 shows an example of a mixture of two samples ($p_{\mathrm{C}} + p_{\mathrm{N}} = 1$) and an example of a mixture of three samples (with the third sample constituting 7.5% of the mixture, $p_{\mathrm{C}} + p_{\mathrm{N}} + 0.075 = 1$). It illustrates how the $e_{\mathrm{EMD,1}}$, $e_{\mathrm{EMD,2}}$ and e_{KDE} errors could be used to test the assumption that the mixture is composed of only two samples. In the first case where the mixture is composed of just two samples, the observed $e_{\mathrm{EMD,1}}$, $e_{\mathrm{EMD,2}}$ and e_{KDE} values are small, and when compared with the 100,000 bootstrapped error values, they indicated that there is a high chance: p=0.19 ($e_{\mathrm{EMD,1}}$), p=0.95 ($e_{\mathrm{EMD,2}}$) and p=0.96 (e_{KDE}) of observing them due to the sampling error in the mixture sample. In the second case where the mixture is composed of three samples, comparison of the observed $e_{\mathrm{EMD,1}}$, $e_{\mathrm{EMD,2}}$ and e_{KDE} values with the bootstrapped values shows that they are unlikely to be a result of the sampling error: p=0.0001 ($e_{\mathrm{EMD,1}}$), p<1e-5 ($e_{\mathrm{EMD,2}}$) and p=0.003 (e_{KDE}). In fact, the value of $e_{\mathrm{EMD,2}}$ is smaller than any of the bootstrapped error values. However, the figure shows only one particular example and the performance of the methods will depend on the mixture composition (contribution of the other sample) and features of the reference and the other samples.

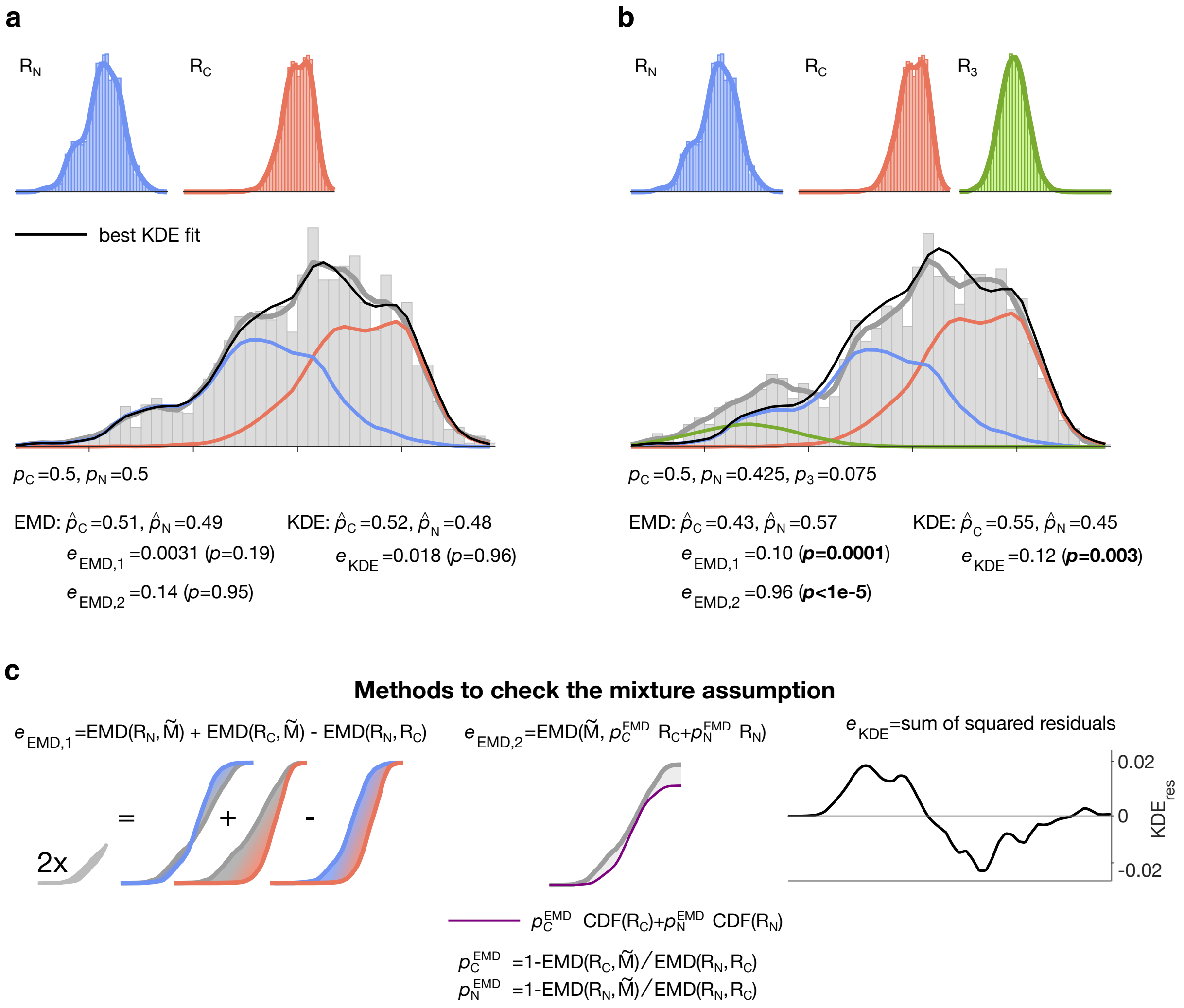

Supplementary Figure 10: Worked examples of checking the mixture assumption, $p_C + p_N = 1$. (a) An example of a mixture that consists of samples from two reference distributions ($p_C = 0.5$, $p_N = 0.5$). The reference distributions are the T1DGRS from the WTCCC dataset. (b) An example of a mixture that consists of samples from three reference distributions. Sample from the third reference distribution (green) has a small contribution ($p_C = 0.5$, $p_N = 0.425$, $p_3 = 0.075$; the reference distribution R_3 is a truncated normal distribution with mean 0.17 and std 0.025). (c) Illustration of the methods for checking the mixture assumption: $e_{EMD,1}$, deviation from collinearity between the two reference distributions and the mixture; $e_{EMD,2}$, EMD between the mixture and a model CDF based on the two independently estimated prevalence values; e_{KDE}, the sum of squared residuals of the final fit.

References

1. Manuel DG, Rosella LC, Stukel TA. Importance of accurately identifying disease in studies using electronic health records. BMJ. 2010;341:c4226.
2. Oram RA, Patel K, Hill A, Shields B, McDonald TJ, Jones A, et al. A Type 1 Diabetes Genetic Risk Score Can Aid Discrimination Between Type 1 and Type 2 Diabetes in Young Adults. Diabetes care. 2015;39(3):337-44.
3. Ntalla I, Kanoni S, Zeng L, Giannakopoulou O, Danesh J, Watkins H, et al. Genetic Risk Score for Coronary Disease Identifies Predispositions to Cardiovascular and Noncardiovascular Diseases. Journal of the American College of Cardiology. 2019;73(23):2932-42.
4. Gao XR, Huang H, Kim H. Polygenic Risk Score Is Associated With Intraocular Pressure and Improves Glaucoma Prediction in the UK Biobank Cohort. Transl Vis Sci Technol. 2019;8(2):10.
5. Thomas NJ, Jones SE, Weedon MN, Shields BM, Oram RA, Hattersley AT. Frequency and phenotype of type 1 diabetes in the first six decades of life: a cross-sectional, genetically stratified survival analysis from UK Biobank. Lancet Diabetes Endocrinol. 2018;6(2):122-9.
6. Manly BFJa. Randomization, Bootstrap and Monte Carlo Methods in Biology. Third edition. ed.
7. Davison AC, Hinkley DV. Bootstrap methods and their application. Cambridge: Cambridge University Press; 1997.
8. Lebwohl B, Sanders DS, Green PHR. Coeliac disease. Lancet. 2018;391(10115):70-81.
9. Smith GD, Ebrahim S. 'Mendelian randomization': can genetic epidemiology contribute to understanding environmental determinants of disease? International journal of epidemiology. 2003;32(1):1-22.
10. Davies NM, Holmes MV, Davey Smith G. Reading Mendelian randomisation studies: a guide, glossary, and checklist for clinicians. BMJ. 2018;362:k601.
11. Hesterberg TC. What Teachers Should Know About the Bootstrap: Resampling in the Undergraduate Statistics Curriculum. The American Statistician. 2015;69(4):371-86.
12. Wellcome Trust Case Control C. Genome-wide association study of 14,000 cases of seven common diseases and 3,000 shared controls. Nature. 2007;447(7145):661-78.
13. Trynka G, Hunt KA, Bockett NA, Romanos J, Mistry V, Szperl A, et al. Dense genotyping identifies and localizes multiple common and rare variant association signals in celiac disease. Nature genetics. 2011;43(12):1193-201.
14. Barker JM, Triolo TM, Aly TA, Baschal EE, Babu SR, Kretowski A, et al. Two single nucleotide polymorphisms identify the highest-risk diabetes HLA genotype: potential for rapid screening. Diabetes. 2008;57(11):3152-5.
15. Udler MS, McCarthy MI, Florez JC, Mahajan A. Genetic Risk Scores for Diabetes Diagnosis and Precision Medicine. Endocrine reviews. 2019;40(6):1500-20.
16. Mitchell RT, Sun A, Mayo A, Forgan M, Comrie A, Gillett PM. Coeliac screening in a Scottish cohort of children with type 1 diabetes mellitus: is DQ typing the way forward? Arch Dis Child. 2016;101(3):230-3.
17. Gutierrez-Achury J, Zhernakova A, Pulit SL, Trynka G, Hunt KA, Romanos J, et al. Fine mapping in the MHC region accounts for 18% additional genetic risk for celiac disease. Nature genetics. 2015;47(6):577-8.
18. Levina E, Bickel P, editors. The Earth Mover's distance is the Mallows distance: some insights from statistics. Proceedings Eighth IEEE International Conference on Computer Vision ICCV 2001; 2001 7-14 July 2001.
19. Muskulus M, Verduyn-Lunel S. Wasserstein distances in the analysis of time series and dynamical systems. Physica D: Nonlinear Phenomena. 2011;240(1):45-58.
20. Cohen S, Guibas L. The Earth Mover''s Distance: Lower Bounds and Invariance under Translation. Stanford University; 1997.
21. Freedman D, Diaconis P. On the histogram as a density estimator:L2 theory. Zeitschrift für Wahrscheinlichkeitstheorie und Verwandte Gebiete. 1981;57(4):453-76.
22. Gill P MW, Wright M. The Levenberg-Marquardt Method. §473 in Practical Optimization. 1981:136-7.
