# Supplementary material for: Estimating disease prevalence in large datasets using genetic risk scores
Source: Nat Commun. 2021 Nov 8;12:6441. doi: 10.1038/s41467-021-26501-7 (PMC8575951; doi:10.1038/s41467-021-26501-7)
Supplement: Supplementary file 3 — Reporting Summary [file 41467_2021_26501_MOESM3_ESM.pdf]

nature research

Corresponding author(s): Nicholas Thomas

Last updated by author(s): Mar 12, 2020

Reporting Summary

Nature Research wishes to improve the reproducibility of the work that we publish. This form provides structure for consistency and transparency in reporting. For further information on Nature Research policies, see Authors & Referees and the Editorial Policy Checklist .

Statistics

For all statistical analyses, confirm that the following items are present in the figure legend, table legend, main text, or Methods section.

n/a	Confirmed	
☐	☒	The exact sample size (*n*) for each experimental group/condition, given as a discrete number and unit of measurement
☐	☒	A statement on whether measurements were taken from distinct samples or whether the same sample was measured repeatedly
☐	☒	The statistical test(s) used AND whether they are one- or two-sided<br>*Only common tests should be described solely by name; describe more complex techniques in the Methods section.*
☐	☒	A description of all covariates tested
☐	☒	A description of any assumptions or corrections, such as tests of normality and adjustment for multiple comparisons
☐	☒	A full description of the statistical parameters including central tendency (e.g. means) or other basic estimates (e.g. regression coefficient) AND variation (e.g. standard deviation) or associated estimates of uncertainty (e.g. confidence intervals)
☐	☒	For null hypothesis testing, the test statistic (e.g. *F*, *t*, *r*) with confidence intervals, effect sizes, degrees of freedom and *P* value noted<br>*Give P values as exact values whenever suitable.*
☒	☐	For Bayesian analysis, information on the choice of priors and Markov chain Monte Carlo settings
☒	☐	For hierarchical and complex designs, identification of the appropriate level for tests and full reporting of outcomes
☒	☐	Estimates of effect sizes (e.g. Cohen's *d*, Pearson's *r*), indicating how they were calculated

Our web collection on statistics for biologists contains articles on many of the points above.

Software and code

Policy information about availability of computer code

Data collection	No software was used for data collection
Data analysis	The Distribution Proportion Estimation software (v1.0.0) used to analyse the data was developed and tested in Python 3.8.2 and Matlab release 2020b (that include other algorithms mentioned in the manuscript). The Distribution Proportion Estimation software (v1.0.0) implementing these methods is archived at https://doi.org/10.5281/zenodo.5512651. The code is open-source and available under version-control here: https://github.com/bdevans/DPE.

For manuscripts utilizing custom algorithms or software that are central to the research but not yet described in published literature, software must be made available to editors/reviewers. We strongly encourage code deposition in a community repository (e.g. GitHub). See the Nature Research guidelines for submitting code & software for further information.

Data

Policy information about availability of data

All manuscripts must include a data availability statement. This statement should provide the following information, where applicable:

- Accession codes, unique identifiers, or web links for publicly available datasets
- A list of figures that have associated raw data
- A description of any restrictions on data availability

UK Biobank data can be obtained after completing an online application, see details at http://www.ukbiobank.ac.uk/using-the-resource/

Wellcome Trust Case Control Consortium genotype data can be obtained through by application to the Wellcome Trust Case Control Consortium Data Access Committee. The procedure is described in more detail at https://www.wtccc.org.uk/info/access_to_data_samples.html

Field-specific reporting

Please select the one below that is the best fit for your research. If you are not sure, read the appropriate sections before making your selection.

☒ Life sciences ☐ Behavioural & social sciences ☐ Ecological, evolutionary & environmental sciences

For a reference copy of the document with all sections, see nature.com/documents/nr-reporting-summary-flat.pdf

Life sciences study design

All studies must disclose on these points even when the disclosure is negative.

Sample size	Cohort sizes were limited by the available number of cases in reference datasets. Controls were limited to match case group sizes. These figures are given clearly in the main body of the article and the Methods section.
Data exclusions	The diabetes and coeliac genetic risk scores are only validated in white European individuals so non-European individuals were excluded as allele frequency's can significantly alter between different ethnicitys altering genetic risk scores. No other exclusion criteria were applied.
Replication	Deterministic parts of the methodology, coded independently in Matlab and Python produce exactly the same outputs (checked upto 5 significant digits).
Randomization	n/a as observational clinical data.
Blinding	n/a as observational clinical data.

Reporting for specific materials, systems and methods

We require information from authors about some types of materials, experimental systems and methods used in many studies. Here, indicate whether each material, system or method listed is relevant to your study. If you are not sure if a list item applies to your research, read the appropriate section before selecting a response.

Materials & experimental systems

n/a	Involved in the study	
☒	☐	Antibodies
☒	☐	Eukaryotic cell lines
☒	☐	Palaeontology
☒	☐	Animals and other organisms
☐	☒	Human research participants
☒	☐	Clinical data

Methods

n/a	Involved in the study	
☒	☐	ChIP-seq
☒	☐	Flow cytometry
☒	☐	MRI-based neuroimaging

Human research participants

Policy information about studies involving human research participants

Population characteristics	The UK Biobank is a cohort of British residents between the ages of 37 and 73, recruited to 22 centres at baseline measurement. The mean age of the European-ancestry individuals included in analyses was 57, with 54% of participants being women. More details are provided in the descriptive UK Biobank paper (doi.org/10.1101/166298).<br><br>Wellcome Trust Case Control Consortium (WTCCC). Type 1 diabetes all cases had an age of diagnosis below 17yr and insulin dependence since diagnosis. Type 2 diabetes were classified based on a clinical diagnosis. More details are provided in the descriptive WTCCC paper (doi.org/10.1038/nature05911)
Recruitment	UK Biobank recruited a population cohort of more than 500,000 people aged between 40 and 70 years registered with the UK National Health Service. There is a reported bias in the UK Biobank data towards participants having a higher socioeconomic status than the background population. This would not affect our results or conclusions as does not impact on genetics and therefore the estimates derived but does mean the absolute values of proportions of diseases estimated may have been slightly different in the general population UK Biobank is derived from.
Ethics oversight	as per above

Note that full information on the approval of the study protocol must also be provided in the manuscript.
